# Supplementary material for: Antibodies against SARS-CoV-2 Alpha, Beta, and Gamma Variants in Pregnant Women and Their Neonates under Antenatal Vaccination with Moderna (mRNA-1273) Vaccine
Source: Vaccines (Basel). 2022 Aug 28;10(9):1415. doi: 10.3390/vaccines10091415 (PMC9505142; doi:10.3390/vaccines10091415)
Supplement: Supplementary file 1 [file vaccines-10-01415-s001.zip › vaccines-1823644-supplementary.pdf]

Table S1. SRBD IgG binding ratio for alpha, beta, and gamma variants in different groups.

Mean	Total	Group A (N=8)	Group B (N=8)	Group C (N=8)	Group D (N=8)	Group E (N=2)	Group F (N=5)
Alpha							
Maternal (%)	63.95	70.84	67.56	68.93	85.57	28.56	18.78
Neonate (%)	72.14	83.50	79.72	80.33	89.75	34.88	15.42
Ratio	1.18	1.26	1.24	1.27	1.05	1.22	0.82
Beta							
Maternal (%)	47.91	54.75	53.98	41.36	58.09	5.62	38.38
Neonate (%)	56.78	67.15	61.71	55.20	61.23	13.76	44.97
Ratio	1.52	1.29	1.36	2.55	1.12	1.25	1.15
Gamma							
Maternal (%)	43.48	55.04	46.38	43.52	53.36	15.48	15.66
Neonate (%)	53.66	67.88	62.33	56.49	62.81	19.74	11.43
Ratio	1.54	1.37	1.84	1.76	1.51	1.22	1.17
P value							
Maternal	**0.0001**	**0.008**	**0.018**	**0.010**	**0.008**	0.658	0.118
Neonate	**0.006**	0.116	0.075	0.068	**0.006**	0.795	**0.040**
Ratio	**0.050**	0.863	0.371	0.250	0.138	0.661	0.139

Words in bold: p value with significance.

Table S2. SRBD IgG binding ratio for SARS-CoV-2 alpha, beta, and gamma variants with different vaccine combinations.

		**Alpha type**			**Beta type**			**Gamma type**		
		Arm 1	Arm 2	p value	Arm 1	Arm 2	p value	Arm 1	Arm 2	p value
Arm 1: 2 COVID-19 + Tdap + Flu<br>Arm 2: 2 COVID-19	N	8	8		8	8		8	8	
	MP (%)	70.84	85.57	0.109	54.75	58.09	0.770	55.04	53.35	0.901
	CB (%)	83.50	89.74	0.208	67.15	61.23	0.547	67.88	62.81	0.634
	Ratio	1.26	1.05	0.113	1.29	1.11	0.327	1.37	1.51	0.681
Arm 1: 1-2 COVID-19 + Tdap + Flu<br>Arm 2: 1-2 COVID-19	N	8	13		8	13		8	13	
	MP (%)	70.84	59.88	0.416	54.75	50.51	0.669	55.04	38.86	0.216
	CB (%)	83.50	61.16	0.082	67.15	54.98	0.199	67.88	43.04	0.060
	Ratio	1.26	1.01	0.062	1.29	1.13	0.346	1.37	1.38	0.986
Arm 1: 2 COVID-19 + Tdap + Flu<br>Arm 2: 2 COVID-19 + Tdap	N	8	8		8	8		8	8	
	MP (%)	70.84	67.56	0.764	54.75	53.98	0.946	55.04	46.38	0.493
	CB (%)	83.50	79.73	0.563	67.15	61.71	0.537	67.88	62.33	0.586
	Ratio	1.26	1.24	0.906	1.29	1.36	0.813	1.37	1.84	0.383
Arm 1: 2 COVID-19 + Tdap + Flu<br>Arm 2: 2 COVID-19 + Flu	N	8	8		8	8		8	8	
	MP (%)	70.84	68.93	0.855	54.75	41.37	0.344	55.04	43.52	0.388
	CB (%)	83.50	80.33	0.546	67.15	55.20	0.317	67.88	56.49	0.359
	Ratio	1.26	1.27	0.935	1.29	2.55	0.165	1.37	1.76	0.406
Arm 1: 2 COVID-19 + Tdap<br>Arm 2: 2 COVID-19	N	8	8		8	8		8	8	
	MP (%)	67.56	85.57	0.051	53.98	58.09	0.746	46.38	53.36	0.627
	CB (%)	79.73	89.75	0.088	61.71	61.23	0.957	62.33	62.81	0.965
	Ratio	1.24	1.05	0.070	1.36	1.12	0.293	1.84	1.51	0.567

Arm 1: 1 COVID-19 + Tdap<br>Arm 2: 1 COVID-19	N	2	5		2	5		2	5	
	MP (%)	28.56	18.78	0.832	5.62	38.38	**0.029**	15.49	15.66	0.989
	CB (%)	34.88	15.43	0.492	13.76	44.97	0.114	19.75	11.43	0.466
	Ratio	1.22	0.82	0.048	1.25	1.15	0.865	1.22	1.17	0.941
Arm 1: 2 COVID-19 + Tdap<br>Arm 2: 1 COVID-19 + Tdap	N	8	2		8	2		8	2	
	MP (%)	67.56	28.56	0.077	53.98	5.62	**0.032**	46.38	15.49	0.159
	CB (%)	79.73	34.88	0.418	61.71	13.76	**0.003**	62.33	19.75	**0.032**
	Ratio	1.24	1.22	0.948	1.36	1.25	0.863	1.84	1.22	0.566
Arm 1: 2 COVID-19 + Flu<br>Arm 2: 2 COVID-19	N	8	8		8	8		8	8	
	MP (%)	68.93	85.57	**0.044**	41.37	58.09	0.269	43.52	53.36	0.511
	CB (%)	80.33	89.75	**0.022**	55.20	61.23	0.613	56.49	62.81	0.627
	Ratio	1.27	1.05	0.201	2.55	1.12	0.116	1.76	1.51	0.622
Arm 1: 2 COVID-19<br>Arm 2: 1 COVID-19	N	8	5		8	5		8	5	
	MP (%)	85.57	18.78	**0.006**	58.09	38.38	0.093	53.36	15.66	**0.011**
	CB (%)	89.75	15.43	**0.002**	61.23	44.97	0.189	62.81	11.43	**0.001**
	Ratio	1.05	0.82	**0.001**	1.12	1.15	0.895	1.51	1.17	0.504
Arm 1: 2 COVID-19 + Tdap<br>Arm 2: 2 COVID-19 + Flu	N	8	8		8	8		8	8	
	MP (%)	67.56	68.93	0.895	53.98	41.37	0.403	46.38	43.52	0.837
	CB (%)	79.73	80.33	0.918	61.71	55.20	0.558	62.33	56.49	0.643
	Ratio	1.24	1.27	0.859	1.36	2.55	0.187	1.84	1.76	0.910

MP, maternal plasma; CB, cord blood; COVID-19, COVID-19 vaccines; Tdap, tetanus toxoid, reduced diphtheria toxoid, and acellular pertussis vaccine; Flu, influenza vaccine; Words in bold: p value with significance.

Table S3. SRBD IgG binding ratio for SARS-CoV-2 alpha, beta, and gamma variants with different vaccines combinations and 2 COVID-19 vaccine interval of 4 weeks.

		**Alpha type**			**Beta type**			**Gamma type**		
Interval 4 weeks		Arm 1	Arm 2	p value	Arm 1	Arm 2	p value	Arm 1	Arm 2	p value
Arm 1: 2 COVID-19 + Tdap + Flu<br>Arm 2: 2 COVID-19	N	3	1		3	1		3	1	
	MP (%)	61.47	71.88	0.729	46.95	78.18	0.132	48.06	65.59	0.580
	CB (%)	77.98	82.26	0.811	57.45	82.43	0.479	65.18	66.47	0.952
	Ratio	1.34	1.14	0.657	1.20	1.05	0.789	1.51	1.01	0.457
Arm 1: 2 COVID-19 + Tdap + Flu<br>Arm 2: 2 COVID-19 + Tdap	N	3	3		3	3		3	3	
	MP (%)	61.47	66.93	0.806	46.95	61.06	0.497	48.06	66.63	0.276
	CB (%)	77.98	82.53	0.718	57.45	71.49	0.441	65.18	79.70	0.253
	Ratio	1.34	1.33	0.976	1.20	1.38	0.709	1.51	1.21	0.356
Arm 1: 2 COVID-19 + Tdap + Flu<br>Arm 2: 2 COVID-19 + Flu	N	3	2		3	2		3	2	
	MP (%)	61.47	84.59	0.268	46.95	5.77	**0.017**	48.06	10.99	0.131
	CB (%)	77.98	91.07	0.290	57.45	30.25	0.302	65.18	27.89	0.068
	Ratio	1.34	1.08	0.362	1.20	5.92	0.129	1.51	3.31	0.396
Arm 1: 2 COVID-19 + Tdap<br>Arm 2: 2 COVID-19	N	3	1		3	1		3	1	
	MP (%)	66.93	71.88	0.893	61.06	78.18	0.678	66.63	65.59	0.940
	CB (%)	82.53	82.26	0.989	71.49	82.43	0.553	79.70	66.47	0.323
	Ratio	1.33	1.14	0.689	1.38	1.05	0.704	1.21	1.01	0.400
Arm 1: 2 COVID-19 + Flu<br>Arm 2: 2 COVID-19	N	2	1		2	1		2	1	
	MP (%)	84.59	71.88	0.282	5.77	78.18	0.055	10.99	65.59	0.128
	CB (%)	91.07	82.26	0.144	30.25	82.43	0.299	27.89	66.47	0.185
	Ratio	1.08	1.14	0.426	5.92	1.05	0.233	3.31	1.01	0.499

Arm 1: 2 COVID-19 +	N	3	2		3	2		3	2	
Tdap	MP (%)	66.93	84.59	0.464	61.06	5.77	0.097	66.63	10.99	**0.009**
Arm 2: 2 COVID-19 +	CB (%)	82.53	91.07	0.505	71.49	30.25	0.073	79.70	27.89	**0.008**
Flu	Ratio	1.33	1.08	0.402	1.38	5.92	0.118	1.21	3.31	0.357

MP, maternal plasma; CB, cord blood; COVID-19, COVID-19 vaccines; Tdap, tetanus toxoid, reduced diphtheria toxoid, and acellular pertussis vaccine; Flu, influenza vaccine; Words in bold: p value with significance.

Table S4. SRBD IgG binding ratio for SARS-CoV-2 alpha, beta, and gamma variants with different vaccines combinations and 2 COVID-19 vaccine interval of 4 – 6 weeks.

		**Alpha type**			**Beta type**			**Gamma type**		
Interval 4 – 6 weeks		Arm 1	Arm 2	p value	Arm 1	Arm 2	p value	Arm 1	Arm 2	p value
Arm 1: 2 COVID-19 + Tdap + Flu<br>Arm 2: 2 COVID-19	N	2	3		2	3		2	3	
	MP (%)	85.82	85.84	0.999	65.12	49.06	0.524	66.77	49.82	0.550
	CB (%)	93.82	88.25	0.413	73.92	56.09	0.429	72.71	58.04	0.461
	Ratio	1.10	1.03	0.550	1.15	1.19	0.771	1.07	1.46	0.551
Arm 1: 2 COVID-19 +	N	2	5		2	5		2	5	
Tdap + Flu	MP (%)	85.82	67.95	0.301	65.12	49.72	0.482	66.77	34.23	0.154
Arm 2: 2 COVID-19 +	CB (%)	93.82	78.05	0.083	73.92	55.85	0.254	72.71	51.91	0.271
Tdap	Ratio	1.10	1.19	0.601	1.15	1.35	0.684	1.07	2.21	0.410
Arm 1: 2 COVID-19 +	N	2	5		2	5		2	5	
Tdap + Flu	MP (%)	85.82	63.66	0.256	65.12	51.09	0.606	66.77	51.28	0.452
Arm 2: 2 COVID-19 +	CB (%)	93.82	75.86	**0.019**	73.92	60.22	0.537	72.71	63.06	0.686
Flu	Ratio	1.10	1.35	0.597	1.15	1.46	0.646	1.07	1.26	0.350
Arm 1: 2 COVID-19 + Tdap<br>Arm 2: 2 COVID-19	N	5	3		5	3		5	3	
	MP (%)	67.95	85.84	0.190	49.72	49.06	0.971	34.23	49.82	0.481
	CB (%)	78.05	88.25	0.227	55.85	56.09	0.984	51.91	58.03	0.651
	Ratio	1.19	1.03	0.211	1.35	1.19	0.681	2.21	1.46	0.501
Arm 1: 2 COVID-19 + Flu<br>Arm 2: 2 COVID-19	N	5	3		5	3		5	3	
	MP (%)	63.66	85.84	0.154	51.09	49.06	0.928	51.28	49.82	0.946
	CB (%)	75.86	88.25	**0.045**	60.22	56.09	0.818	63.06	58.04	0.784
	Ratio	1.35	1.03	0.390	1.46	1.19	0.613	1.26	1.46	0.702
Arm 1: 2 COVID-19 +	N	5	5		5	5		5	5	
Tdap	MP (%)	67.95	63.66	0.753	49.72	51.09	0.940	34.23	51.28	0.319
Arm 2: 2 COVID-19 +	CB (%)	78.05	75.86	0.766	55.85	60.22	0.743	51.91	63.06	0.473
Flu	Ratio	1.19	1.35	0.563	1.35	1.46	0.820	2.21	1.26	0.248

MP, maternal plasma; CB, cord blood; COVID-19, COVID-19 vaccines; Tdap, tetanus toxoid, reduced diphtheria toxoid, and acellular pertussis vaccine; Flu, influenza vaccine; Words in bold: p value with significance.

Table S5. SRBD IgG binding ratio for SARS-CoV-2 alpha, beta, and gamma variants with different vaccines combinations and 2 COVID-19 vaccine interval of > 6 weeks.

		**Alpha type**			**Beta type**			**Gamma type**		
Interval > 6 weeks		Arm 1	Arm 2	p value	Arm 1	Arm 2	p value	Arm 1	Arm 2	p value
Arm 1: 2 COVID-19 + Tdap + Flu<br>Arm 2: 2 COVID-19	N	2	3		2	3		2	3	
	MP (%)	59.41	87.73	0.355	68.27	65.29	0.919	67.52	57.51	0.798
	CB (%)	75.76	92.45	**0.028**	74.34	59.33	0.516	70.35	69.77	0.987
	Ratio	1.39	1.06	0.545	1.12	0.97	0.543	1.03	1.78	0.451

Arm 1: 2 COVID-19 + Tdap + Flu<br>Arm 2: 2 COVID-19 + Tdap	N	2			2			2		
	MP (%)	59.41	0	-	68.27	0	-	67.52	0	-
	CB (%)	75.76			74.34			70.35		
	Ratio	1.39			1.12			1.03		
Arm 1: 2 COVID-19 + Tdap + Flu<br>Arm 2: 2 COVID-19 + Flu	N	2	1		2	1		2	1	
	MP (%)	59.41	63.96	0.910	68.27	63.95	0.900	67.52	69.73	0.964
	CB (%)	75.76	81.19	0.436	74.34	79.99	0.793	70.35	80.86	0.848
	Ratio	1.39	1.27	0.882	1.12	1.25	0.621	1.03	1.16	0.188
Arm 1: 2 COVID-19 + Tdap<br>Arm 2: 2 COVID-19	N		3			3			3	
	MP (%)	0	87.73	-	0	65.29	-	0	57.51	-
	CB (%)		92.45			59.33			69.77	
	Ratio		1.06			0.97			1.78	
Arm 1: 2 COVID-19 + Flu<br>Arm 2: 2 COVID-19	N	1	3		1	3		1	3	
	MP (%)	63.96	87.73	0.147	63.95	65.29	0.975	69.73	57.51	0.826
	CB (%)	81.19	92.45	0.187	79.99	59.33	0.558	80.86	69.77	0.819
	Ratio	1.27	1.06	0.183	1.25	0.97	0.447	1.16	1.78	0.688
Arm 1: 2 COVID-19 + Tdap<br>Arm 2: 2 COVID-19 + Flu	N		1			1			1	
	MP (%)	0	63.96	-	0	63.95	-	0	69.73	-
	CB (%)		81.19			79.99			80.86	
	Ratio		1.27			1.25			1.16	

MP, maternal plasma; CB, cord blood; COVID-19, COVID-19 vaccines; Tdap, tetanus toxoid, reduced diphtheria toxoid, and acellular pertussis vaccine; Flu, influenza vaccine; Words in bold: p value with significance.

Table S6. SRBD IgG binding ratio for SARS-CoV-2 alpha, beta, and gamma variants with different intervals from 2nd COVID-19 vaccine dose to childbirth.

2 COVID-19 vaccines		Alpha type						Beta type					
Interval between last dose to delivery		Arm 1	Arm 2	Arm 3	Arm 4	Arm 5	p value	Arm 1	Arm 2	Arm 3	Arm 4	Arm 5	p value
Arm 1: ≤ 6 wks<br>Arm 2: 6 – 8 wks<br>Arm 3: 8 – 10 wks<br>Arm 4: 10 – 12 wks<br>Arm 3: > 12 wks	N	5	6	5	8	6		5	6	5	8	6	
	MP (%)	85.17	70.24	60.05	61.62	86.48	**0.023**	73.86	62.86	50.34	42.42	42.35	0.164
	CB (%)	87.40	79.75	76.79	79.20	90.79	0.109	70.13	68.21	62.95	55.76	52.02	0.515
	Ratio	1.03	1.17	1.42	1.37	1.05	0.088	0.98	1.11	1.49	1.72	2.36	0.418
		Gamma type											
Interval between last dose to delivery		Arm 1	Arm 2	Arm 3	Arm 4	Arm 5	p value						

Arm 1: ≤ 6 ks<br>Arm 2: 6 – 8 wks<br>Arm 3: 8 – 10 wks<br>Arm 4: 10 – 12 wks<br>Arm 3: > 12 wks	N	5	6	5	8	6	
	MP (%)	66.87	58.22	45.59	51.07	33.22	0.284
	CB (%)	73.57	68.17	64.28	58.54	52.59	0.591
	Ratio	1.12	1.28	1.72	1.29	2.67	**0.042**

MP, maternal plasma; CB, cord blood; wks, weeks; Words in bold: p value with significance.

Table S7. SRBD IgG binding ratio for SARS-CoV-2 alpha, beta, and gamma variants with different vaccines combinations and a ≤ 6-week interval between the 2nd dose of COVID-19 vaccine and childbirth.

		**Alpha type**			**Beta type**			**Gamma type**		
Interval ≤ 6 weeks		Arm 1	Arm 2	p value	Arm 1	Arm 2	p value	Arm 1	Arm 2	p value
Arm 1: 2 COVID-19 + Tdap + Flu<br>Arm 2: 2 COVID-19	N	1	3		1	3		1	3	
	MP (%)	95.76	87.88	0.588	83.19	69.72	0.667	72.45	69.67	0.929
	CB (%)	97.32	89.52	0.516	90.62	64.21	0.183	90.37	71.96	0.504
	Ratio	1.02	1.02	0.897	1.09	0.98	0.775	1.25	1.06	0.436
Arm 1: 2 COVID-19 + Tdap + Flu<br>Arm 2: 2 COVID-19 + Tdap	N	1	1		1	1		1	1	
	MP (%)	95.76	66.45	-	83.19	76.93	-	72.45	52.89	-
	CB (%)	97.32	71.11		90.62	67.39		90.37	61.59	
	Ratio	1.02	1.07		1.09	0.88		1.25	1.16	
Arm 1: 2 COVID-19 + Tdap + Flu<br>Arm 2: 2 COVID-19 + Flu	N	1			1			1		
	MP (%)	95.76	0	-	83.19	0	-	72.45	0	-
	CB (%)	97.32			90.62			90.37		
	Ratio	1.02			1.09			1.25		
Arm 1: 2 COVID-19 + Tdap<br>Arm 2: 2 COVID-19	N	1	3		1	3		1	3	
	MP (%)	66.45	87.88	0.224	76.93	69.72	0.814	52.89	69.67	0.604
	CB (%)	71.11	89.52	0.206	67.39	64.21	0.832	61.59	71.96	0.693
	Ratio	1.07	1.02	0.255	0.88	0.98	0.780	1.16	1.06	0.647
Arm 1: 2 COVID-19 + Flu<br>Arm 2: 2 COVID-19	N		3			3			3	
	MP (%)	0	87.88	-	0	69.72	-	0	69.67	-
	CB (%)		89.52			64.21			71.96	
	Ratio		1.02			0.98			1.06	
Arm 1: 2 COVID-19 + Tdap<br>Arm 2: 2 COVID-19 + Flu	N	1			1			1		
	MP (%)	66.45	0	-	76.93	0	-	52.89	0	-
	CB (%)	71.11			67.39			61.59		
	Ratio	1.07			0.88			1.16		

MP, maternal plasma; CB, cord blood; COVID-19, COVID-19 vaccines; Tdap, tetanus toxoid, reduced diphtheria toxoid, and acellular pertussis vaccine; Flu, influenza vaccine.

Table S8. SRBD IgG binding ratio for SARS-CoV-2 alpha, beta, and gamma variants with different vaccines combinations and a 6 – 8-week interval between the 2nd dose of COVID-19 vaccine and childbirth.

		**Alpha type**			**Beta type**			**Gamma type**		

Interval 6 – 8 weeks		Arm 1	Arm 2	p value	Arm 1	Arm 2	p value	Arm 1	Arm 2	p value
Arm 1: 2 COVID-19 + Tdap + Flu<br>Arm 2: 2 COVID-19	N	1	2		1	2		1	2	
	MP (%)	77.90	75.82	0.811	84.03	77.71	0.081	90.13	74.52	0.497
	CB (%)	78.33	87.54	0.498	84.05	83.31	0.715	95.22	79.52	0.613
	Ratio	1.01	1.15	0.071	1.00	1.07	0.257	1.06	1.06	0.963
Arm 1: 2 COVID-19 + Tdap + Flu<br>Arm 2: 2 COVID-19 + Tdap	N	1	1	-	1	1	-	1	1	-
	MP (%)	77.90	43.25		84.03	57.75		90.13	28.09	
	CB (%)	78.33	64.61		84.04	58.28		95.22	58.56	
	Ratio	1.01	1.49		1.00	1.01		1.06	2.08	
Arm 1: 2 COVID-19 + Tdap + Flu<br>Arm 2: 2 COVID-19 + Flu	N	1	2		1	2		1	2	
	MP (%)	77.90	74.33	0.875	84.03	39.97	0.481	90.13	41.03	0.504
	CB (%)	78.33	80.24	0.456	84.05	50.14	0.631	95.22	48.09	0.559
	Ratio	1.01	1.10	0.793	1.00	1.26	**0.037**	1.06	1.20	0.298
Arm 1: 2 COVID-19 + Tdap<br>Arm 2: 2 COVID-19	N	1	2		1	2		1	2	
	MP (%)	43.25	75.82	0.132	57.75	77.71	**0.026**	28.01	74.52	0.205
	CB (%)	64.61	87.54	0.241	58.28	83.31	**0.039**	58.56	79.52	0.524
	Ratio	1.49	1.15	**0.031**	1.01	1.07	0.289	2.08	1.06	0.051
Arm 1: 2 COVID-19 + Flu<br>Arm 2: 2 COVID-19	N	2	2		2	2		2	2	
	MP (%)	74.33	75.82	0.905	39.97	77.71	0.256	41.03	74.52	0.381
	CB (%)	80.24	87.54	0.307	50.14	83.31	0.382	48.09	79.52	0.467
	Ratio	1.10	1.15	0.788	1.26	1.07	**0.011**	1.20	1.06	0.159
Arm 1: 2 COVID-19 + Tdap<br>Arm 2: 2 COVID-19 + Flu	N	1	2		1	2		1	2	
	MP (%)	43.25	74.33	0.334	57.75	39.97	0.742	28.09	41.03	0.838
	CB (%)	64.61	80.24	0.067	58.28	50.14	0.901	58.56	48.09	0.884
	Ratio	1.49	1.10	0.405	1.01	1.26	**0.038**	2.08	1.20	0.053

MP, maternal plasma; CB, cord blood; COVID-19, COVID-19 vaccines; Tdap, tetanus toxoid, reduced diphtheria toxoid, and acellular pertussis vaccine; Flu, influenza vaccine; Words in bold: p value with significance.

Table S9. SRBD IgG binding ratio for SARS-CoV-2 alpha, beta, and gamma variants with different vaccines combinations and an 8 – 10-week interval between the 2nd dose of COVID-19 vaccine and childbirth.

		**Alpha type**			**Beta type**			**Gamma type**		
Interval 8 – 10 weeks		Arm 1	Arm 2	p value	Arm 1	Arm 2	p value	Arm 1	Arm 2	p value
Arm 1: 2 COVID-19 + Tdap + Flu<br>Arm 2: 2 COVID-19	N	1	1	-	1	1	-	1	1	-
	MP(%)	55.04	86.09		59.49	28.67		52.19	8.73	
	CB (%)	67.84	87.34		76.96	32.94		76.09	27.14	
	Ratio	1.23	1.01		1.29	1.15		1.46	3.11	
Arm 1: 2 COVID-19 + Tdap + Flu<br>Arm 2: 2 COVID-19 + Tdap	N	1	1	-	1	1	-	1	1	-
	MP(%)	55.04	65.81		59.49	68.34		52.19	69.44	
	CB (%)	67.84	84.00		76.96	71.77		76.09	73.69	
	Ratio	1.23	1.28		1.29	1.05		1.46	1.06	
Arm 1: 2 COVID-19 + Tdap + Flu<br>Arm 2: 2 COVID-19 + Flu	N	1	2		1	2		1	2	
	MP(%)	55.04	46.67	0.845	59.49	47.61	0.853	52.19	48.78	0.163
	CB (%)	67.84	72.40	0.799	79.96	66.55	0.716	76.09	72.23	0.463
	Ratio	1.23	1.79	0.674	1.29	1.99	0.748	1.46	1.48	0.698

Arm 1: 2 COVID-19 + Tdap	N	1	1		1	1		1	1	
Arm 2: 2 COVID-19	MP(%)	65.81	86.09	-	68.34	28.67	-	69.44	8.73	-
	CB (%)	84.00	87.34		71.77	32.94		73.69	27.14	
	Ratio	1.28	1.01		1.05	1.15		1.06	3.11	
Arm 1: 2 COVID-19 + Flu	N	2	1		2	1		2	1	
Arm 2: 2 COVID-19	MP(%)	46.67	86.09	0.450	47.61	28.67	0.772	48.78	8.73	**0.014**
	CB (%)	72.40	87.34	0.478	66.55	32.94	0.365	72.23	27.14	**0.048**
	Ratio	1.79	1.01	0.578	1.99	1.15	0.702	1.48	3.11	**0.017**
Arm 1: 2 COVID-19 +	N	1	2		1	2		1	2	
Tdap	MP(%)	65.81	46.67	0.671	68.34	47.61	0.753	69.44	48.78	**0.027**
Arm 2: 2 COVID-19 +	CB (%)	84.00	72.40	0.559	71.77	66.55	0.850	73.69	72.23	0.743
Flu	Ratio	1.28	1.79	0.696	1.05	1.99	0.673	1.06	1.48	0.066

MP, maternal plasma; CB, cord blood; COVID-19, COVID-19 vaccines; Tdap, tetanus toxoid, reduced diphtheria toxoid, and acellular pertussis vaccine; Flu, influenza vaccine; Words in bold: p value with significance.

Table S10. SRBD IgG binding ratio for SARS-CoV-2 alpha, beta, and gamma variants with different vaccines combinations and a 10 – 12-week interval between the 2nd dose of COVID-19 vaccine and childbirth.

		**Alpha type**			**Beta type**			**Gamma type**		
Interval 10 – 12 weeks		Arm 1	Arm 2	p value	Arm 1	Arm 2	p value	Arm 1	Arm 2	p value
Arm 1: 2 COVID-19 + Tdap + Flu	N	4			4			4		
Arm 2: 2 COVID-19	MP (%)	61.54	0	-	45.22	0	-	49.49	0	-
	CB (%)	82.40			54.31			54.99		
	Ratio	1.44			1.19			1.24		
Arm 1: 2 COVID-19 +	N	4	2		4	2		4	2	
Tdap + Flu	MP (%)	61.54	47.49	0.378	45.22	42.81	0.902	49.49	61.52	0.487
Arm 2: 2 COVID-19 +	CB (%)	82.40	67.21	0.070	54.31	58.65	0.753	54.99	71.55	0.176
Tdap	Ratio	1.44	1.46	0.959	1.19	1.57	0.420	1.24	1.18	0.888
Arm 1: 2 COVID-19 +	N	4	2		4	2		4	2	
Tdap + Flu	MP (%)	61.54	75.90	0.458	45.22	36.43	0.800	49.49	43.76	0.809
Arm 2: 2 COVID-19 +	CB (%)	82.40	84.81	0.812	54.31	55.79	0.923	54.99	52.64	0.881
Flu	Ratio	1.44	1.13	0.193	1.19	2.94	0.525	1.24	1.49	0.627
Arm 1: 2 COVID-19 + Tdap	N	2			2			2		
Arm 2: 2 COVID-19	MP (%)	47.49	0	-	42.81	0	-	61.53	0	-
	CB (%)	67.21			58.65			71.55		
	Ratio	1.46			1.57			1.18		
Arm 1: 2 COVID-19 + Flu	N	2			2			2		
Arm 2: 2 COVID-19	MP (%)	75.90	0	-	36.43	0	-	43.76	0	-
	CB (%)	84.81			55.79			52.64		
	Ratio	1.13			2.94			1.49		
Arm 1: 2 COVID-19 +	N	2	2		2	2		2	2	
Tdap	MP (%)	47.49	75.90	0.191	42.81	36.43	0.857	61.53	43.76	0.581
Arm 2: 2 COVID-19 +	CB (%)	67.21	84.81	0.142	58.65	55.79	0.806	71.55	52.64	0.415
Flu	Ratio	1.46	1.13	0.326	1.57	2.94	0.561	1.18	1.49	0.612

MP, maternal plasma; CB, cord blood; COVID-19, COVID-19 vaccines; Tdap, tetanus toxoid, reduced diphtheria toxoid, and acellular pertussis vaccine; Flu, influenza vaccine.

Table S11. SRBD IgG binding ratio for SARS-CoV-2 alpha, beta, and gamma variants with different vaccines combinations and a > 12-week interval between the 2nd dose of COVID-19 vaccine and childbirth.

		Alpha type			Beta type			Gamma type		
Interval > 12 weeks		Arm 1	Arm 2	p value	Arm 1	Arm 2	p value	Arm 1	Arm 2	p value
Arm 1: 2 COVID-19 + Tdap + Flu Arm 2: 2 COVID-19	N		1			1			1	
	MP (%)	0	91.21	-	0	27.98	-	0	20.19	-
	CB (%)		93.36			36.50			47.82	
	Ratio		1.02			1.30			2.29	
Arm 1: 2 COVID-19 + Tdap + Flu Arm 2: 2 COVID-19 + Tdap	N		3			3			3	
	MP (%)	0	90.00	-	0	47.72	-	0	32.53	-
	CB (%)		94.57			59.65			53.90	
	Ratio		1.05			1.60			2.67	
Arm 1: 2 COVID-19 + Tdap + Flu Arm 2: 2 COVID-19 + Flu	N		2			2			2	
	MP (%)	0	78.82	-	0	41.47	-	0	40.49	-
	CB (%)		83.86			48.33			53.01	
	Ratio		1.06			4.01			2.87	
Arm 1: 2 COVID-19 + Tdap Arm 2: 2 COVID-19	N	3	1		3	1		3	1	
	MP (%)	90.00	91.21	0.858	47.72	27.98	0.694	32.53	20.79	0.811
	CB (%)	94.57	93.36	0.712	59.65	36.50	0.539	53.90	47.82	0.894
	Ratio	1.05	1.02	0.673	1.60	1.30	0.746	2.67	2.29	0.894
Arm 1: 2 COVID-19 + Flu Arm 2: 2 COVID-19	N	2	1		2	1		2	1	
	MP (%)	78.82	91.21	0.198	41.47	27.98	0.876	40.49	20.79	0.805
	CB (%)	83.86	93.36	0.531	48.33	36.50	0.872	53.01	47.82	0.940
	Ratio	1.06	1.02	0.707	4.01	1.30	0.694	2.87	2.29	0.882
Arm 1: 2 COVID-19 + Tdap Arm 2: 2 COVID-19 + Flu	N	3	2		3	2		3	2	
	MP (%)	90.00	78.82	0.076	47.72	41.47	0.887	32.53	40.49	0.850
	CB (%)	94.57	83.86	0.315	59.65	48.33	0.748	53.90	53.01	0.981
	Ratio	1.05	1.06	0.852	1.60	4.01	0.569	2.67	2.87	0.930

MP, maternal plasma; CB, cord blood; COVID-19, COVID-19 vaccines; Tdap, tetanus toxoid, reduced diphtheria toxoid, and acellular pertussis vaccine; Flu, influenza vaccine.

Table S12. SRBD IgG binding ratio for SARS-CoV-2 alpha, beta, and gamma variants with different vaccines combinations and a > 6-week interval between the 2nd dose of COVID-19 vaccine and childbirth.

		Alpha type			Beta type			Gamma type		
Interval > 6 weeks		Arm 1	Arm 2	p value	Arm 1	Arm 2	p value	Arm 1	Arm 2	p value
Arm 1: 2 COVID-19 + Tdap + Flu Arm 2: 2 COVID-19	N	6	4		6	4		6	4	
	MP (%)	63.18	82.23	0.071	54.07	53.02	0.950	56.72	44.64	0.526
	CB (%)	79.29	88.94	0.128	63.04	59.02	0.793	65.21	58.49	0.664
	Ratio	1.33	1.09	0.131	1.18	1.15	0.864	1.25	1.88	0.302
Arm 1: 2 COVID-19 + Tdap + Flu Arm 2: 2 COVID-19 + Tdap	N	6	7		6	7		6	7	
	MP (%)	63.18	67.72	0.709	54.07	50.69	0.785	56.72	45.45	0.450
	CB (%)	79.29	80.96	0.817	63.04	60.90	0.832	65.21	62.44	0.818
	Ratio	1.33	1.26	0.673	1.18	1.43	0.351	1.25	1.93	0.294
Arm 1: 2 COVID-19 + Tdap + Flu	N	6	8		6	8		6	8	
	MP (%)	63.18	68.93	0.594	54.07	41.37	0.364	56.72	43.52	0.370

Arm 2: 2 COVID-19 + Flu	CB (%)	79.29	80.33	0.841	63.04	55.20	0.553	65.21	56.49	0.530
	Ratio	1.33	1.27	0.787	1.18	2.55	0.130	1.25	1.76	0.338
Arm 1: 2 COVID-19 + Tdap<br>Arm 2: 2 COVID-19	N	7	4		7	4		7	4	
	MP (%)	67.72	82.23	0.166	50.69	53.02	0.892	45.45	44.64	0.967
	CB (%)	80.96	88.94	0.219	60.90	59.02	0.890	62.44	58.49	0.802
	Ratio	1.26	1.09	0.214	1.43	1.15	0.258	1.93	1.88	0.954
Arm 1: 2 COVID-19 + Flu<br>Arm 2: 2 COVID-19	N	8	4		8	4		8	4	
	MP (%)	68.93	82.23	0.225	41.37	53.02	0.561	43.52	44.64	0.954
	CB (%)	80.33	88.94	0.096	55.20	59.02	0.822	56.49	58.49	0.909
	Ratio	1.27	1.09	0.449	2.55	1.15	0.123	1.76	1.88	0.866
Arm 1: 2 COVID-19 + Tdap<br>Arm 2: 2 COVID-19 + Flu	N	7	8		7	8		7	8	
	MP (%)	67.72	68.93	0.913	50.69	41.37	0.553	45.45	43.52	0.897
	CB (%)	80.96	80.33	0.921	60.90	55.20	0.632	62.44	56.49	0.661
	Ratio	1.26	1.27	0.964	1.43	2.55	0.213	1.93	1.76	0.808

MP, maternal plasma; CB, cord blood; COVID-19, COVID-19 vaccines; Tdap, tetanus toxoid, reduced diphtheria toxoid, and acellular pertussis vaccine; Flu, influenza vaccine.

Table S13. SRBD IgG binding ratio for SARS-CoV-2 alpha, beta, and gamma variants with different vaccines combinations and a ≤ 12-week interval between the 2nd dose of COVID-19 vaccine and childbirth.

		**Alpha type**			**Beta type**			**Gamma type**		
Interval ≤ 12 weeks		Arm 1	Arm 2	p value	Arm 1	Arm 2	p value	Arm 1	Arm 2	p value
Arm 1: 2 COVID-19 + Tdap + Flu<br>Arm 2: 2 COVID-19	N	7	6		7	6		7	6	
	MP (%)	67.84	83.56	0.118	58.23	65.54	0.545	58.96	61.13	0.883
	CB (%)	81.87	88.49	0.228	66.98	65.37	0.888	68.81	67.01	0.889
	Ratio	1.29	1.06	0.121	1.16	1.04	0.355	1.25	1.40	0.668
Arm 1: 2 COVID-19 + Tdap + Flu<br>Arm 2: 2 COVID-19 + Tdap	N	7	5		7	5		7	5	
	MP (%)	67.84	54.09	0.234	58.23	57.73	0.964	58.96	54.69	0.720
	CB (%)	81.87	70.83	0.096	66.98	62.95	0.681	68.81	67.39	0.870
	Ratio	1.29	1.35	0.722	1.16	1.22	0.819	1.25	1.34	0.719
Arm 1: 2 COVID-19 + Tdap + Flu<br>Arm 2: 2 COVID-19 + Flu	N	7	6		7	6		7	6	
	MP (%)	67.84	65.63	0.858	58.23	41.33	0.263	58.96	44.52	0.286
	CB (%)	81.87	79.15	0.651	66.98	57.49	0.444	68.81	57.66	0.407
	Ratio	1.29	1.34	0.822	1.16	2.06	0.215	1.25	1.39	0.486
Arm 1: 2 COVID-19 + Tdap<br>Arm 2: 2 COVID-19	N	5	6		5	6		5	6	
	MP (%)	54.09	83.56	**0.002**	57.73	65.54	0.566	54.69	61.13	0.684
	CB (%)	70.83	88.49	**0.003**	62.95	65.37	0.787	67.39	67.01	0.975
	Ratio	1.35	1.06	0.057	1.22	1.04	0.453	1.34	1.40	0.876
Arm 1: 2 COVID-19 + Flu<br>Arm 2: 2 COVID-19	N	6	6		6	6		6	6	
	MP (%)	65.63	83.56	0.093	41.33	65.54	0.151	44.52	61.13	0.325
	CB (%)	79.15	88.49	0.065	57.49	65.37	0.537	57.66	67.01	0.541
	Ratio	1.34	1.06	0.222	2.06	1.04	0.166	1.39	1.40	0.979
Arm 1: 2 COVID-19 + Tdap<br>Arm 2: 2 COVID-19 + Flu	N	5	6		5	6		5	6	
	MP (%)	54.09	65.63	0.322	57.73	41.33	0.298	54.69	44.52	0.457
	CB (%)	70.83	79.15	0.137	62.95	57.49	0.618	67.39	57.66	0.420
	Ratio	1.35	1.34	0.972	1.22	2.06	0.252	1.34	1.39	0.813

MP, maternal plasma; CB, cord blood; COVID-19, COVID-19 vaccines; Tdap, tetanus toxoid, reduced diphtheria toxoid, and acellular pertussis vaccine; Flu, influenza vaccine; Words in bold: p value with significance.
